# Improving inceptionV4 model based on fractional-order snow leopard optimization algorithm for diagnosing of ACL tears

**DOI:** 10.1038/s41598-024-60419-6

**Published:** 2024-04-29

**Authors:** Delei Wang, Yanqing Yan

**Affiliations:** 1 Zhejiang Pharmaceutical University, Ningbo, 315500 Zhejiang China; 2 Guangdong University of Science and Technology, Dongguan, 523000 Guangdong China

**Keywords:** Anterior Cruciate Ligament tear, InceptionV4 model, Optimized design, Fractional-order Snow Leopard Optimization Algorithm, Knee MRI images, Physiology, Medical research, Engineering, Mathematics and computing

## Abstract

In the current research study, a new method is presented to diagnose Anterior Cruciate Ligament (ACL) tears by introducing an optimized version of the InceptionV4 model. Our proposed methodology utilizes a custom-made variant of the Snow Leopard Optimization Algorithm, known as the Fractional-order Snow Leopard Optimization Algorithm (FO-LOA), to extract essential features from knee magnetic resonance imaging (MRI) images. This results in a substantial improvement in the accuracy of ACL tear detection. By effectively extracting critical features from knee MRI images, our proposed methodology significantly enhances diagnostic accuracy, potentially reducing false negatives and false positives. The enhanced model based on FO-LOA underwent thorough testing using the MRNet dataset, demonstrating exceptional performance metrics including an accuracy rate of 98.00%, sensitivity of 98.00%, precision of 97.00%, specificity of 98.00%, F1-score of 98.00%, and Matthews Correlation Coefficient (MCC) of 88.00%. These findings surpass current methodologies like Convolutional Neural Network (CNN), Inception-v3, Deep Belief Networks and Improved Honey Badger Algorithm (DBN/IHBA), integration of the CNN with an Amended Cooking Training-based Optimizer version (CNN/ACTO), Self-Supervised Representation Learning (SSRL), signifying a significant breakthrough in ACL injury diagnosis. Using FO-SLO to optimize the InceptionV4 framework shows promise in improving the accuracy of ACL tear identification, enabling prompt and efficient treatment interventions.

## Introduction

### Background

The Anterior Cruciate Ligament (ACL) has been a vital band of fibrous tissue that links the femur (thighbone) to the tibia (shinbone) and is a significant contributor to the stability of the knee joint. It provides restraint to forward motion of the shinbone and rotation of the knee^1^. However, it is also a frequently damaged ligament, especially in athletes who take part in sports that necessitate quick pivots, stops, or changes in direction, such as basketball, soccer, tennis, and volleyball^2^. A torn ACL can be a severe injury that results in intense pain, swelling, instability, and limited mobility, and may require surgery and a lengthy rehabilitation process to fully recover^3^. The rehabilitation process may also include physical therapy and exercises to help regain strength, flexibility, and stability in the knee joint^4^. Injuries to the ACL can cause pain, instability, and reduced mobility, and often require surgical intervention to repair or reconstruct the ligament in order to restore function and prevent further damage^5^. The ACL tears are of different kinds^6^.

When a patient suffers an ACL injury, it is crucial to assess the extent of the damage as accurately as possible. This task is typically carried out by a qualified radiologist who has received specialized training in evaluating such injuries^7^. The radiologist's assessment involves a thorough examination of the affected area to determine the severity of the injury. This could be a complete rupture of the ACL, a partial tear (also known as a strain), or no injury to the ACL at all^8^.

In addition to determining the type and severity of the injury, the radiologist's analysis may also include additional details, such as the location of the injury and the potential impact it may have on the patient's overall health and physical abilities^9^. This information is critical in helping to develop an effective treatment plan and ensuring the patient receives the best possible care^10^. Ultimately, the radiologist's expertise plays a vital role in helping patients recover from ACL injuries and return to their normal activities as quickly and safely as possible^11^. Of course, the accuracy of the detection is of utmost importance^12^.

An accurate and reproducible detection of a complete tear of the ACL is crucial for making informed decisions about treatment options^13^. It is important to have a thorough sympathetic of the degree of the damage, as well as the patient's individual circumstances, in order to provide the most effective and appropriate treatment plan^14^. Failure to correctly diagnose a complete ACL tear can lead to delayed treatment and potentially worsening of the condition, which can have long-term consequences for the patient's mobility and quality of life. Therefore, accurate diagnosis is a critical step in ensuring optimal outcomes for patients with a complete ACL tear. There are different image-based tools for detecting the ACL tear.

Medical imaging is an essential tool in diagnosing a wide range of injuries and conditions, and radiologists are highly skilled medical professionals who specialize in interpreting these images. When it comes to identifying and diagnosing Anterior Cruciate Ligament (ACL) tears, radiologists are particularly well-equipped to do so. By analyzing a variety of medical pictures, comprising MRI scans, CT scans, and X-rays, radiologists can identify the subtle changes and irregularities that may indicate an ACL tear. With their expertise and attention to detail, radiologists play a critical role in ensuring that patients receive timely and accurate diagnoses, which can be crucial in guiding treatment decisions and achieving the best possible outcomes. However, the MRI (Magnetic Resonance Imaging) has been considered the best tool for the diagnosis of the tear in ACL.

If someone suffers an ACL injury, the most common method to diagnose it is performing a magnetic resonance imaging (MRI) scan on the knee joint^15–17^. This diagnostic test produces a highly detailed image of the interior constructions of the knee, which is then carefully examined by a radiologist or other trained medical professional^18^. By analyzing these images, the radiologist can identify any signs of damage to the ACL, including tears or other abnormalities^19^. This thorough examination helps to determine the extent of the injury, which is crucial for developing an effective treatment plan^20^. MRI scans are particularly helpful in diagnosing ACL injuries as they provide a clear view of the soft tissues of the knee, which are not visible in X-rays. Moreover, MRI scans do not expose patients to radiation, making them a safer diagnostic tool compared to X-rays and CT scans.

### Related works

There are numerous studies conducted on this particular issue, which will be exhaustively discussed in the following section. These studies provide a comprehensive analysis and insight into the matter, enabling us to better understand the intricacies and complexities involved.

Minamoto et al.^21^ proposed a model in order to assess the ability of a CNN system's accuracy to detect the tears in Anterior Cruciate Ligament (ACL). This was conducted merely by a knee MRI; the results of image got compared with the results of the experienced doctors. Sagittal Magnetic Resonance Images from 100 patients (without /with Anterior Cruciate Ligament harms proved by arthroscopy) were utilized to train a Convolutional Neural Network to determine the possibility of an Anterior Cruciate Ligament tear on an only MRI piece. The CNN's decision was compared to 12 physicians who reviewed the identical images. The negative predictive value, the positive predictive value, the accuracy, the specificity, the sensitivity of the CNN categorization was, in turn, 91.0, 87.0, 88.5, 86.0, and 91.0%. Although the physicians' readings had similar overall values, their specificity was lower compared to the CNN's classification. Consequently, the accuracy of the human readers decreased. in the end, it was concluded that the trained Convolutional Neural Network could diagnose ACL ruptures in a great way with high accuracy in comparison with the accuracy of the physicians, diagnosis.

Sun et al.^22^ suggested a hierarchical method in order to gain more accuracy in detecting ACL tears injury. In the beginning, in order to increase the quality of the image, the pre-processing strategies got applied. Next, by the use of integrated mode of DCT (Discrete Cosine Transform) and GLCM (Co-occurrence Matrix) and the attributes of the pictures were recovered. The DBN (Deep Belief Network) received the features for categorization and was optimized utilizing the Enhanced Honey Badger Algorithm. The outcomes of the suggested study were compared with other approaches, such as Convolutional Neural Networks (CNN), Fuzzy, RF (Random Forest), and Distance and Neural Networks (ED/NN). It was evidenced that the specificity, sensitivity, and accuracy of the suggested method were, in turn, 80%, 98%, and 96%, which illustrated the best efficacy compared to other approaches.

Chen et al.^23^ proposed a model to assess detection efficiency of the transfer learning approach to grade detection of Anterior Cruciate Ligament tear using an enhanced precision locating of thin-piece oblique sagittal FS-PDWI order and juxtapose the diagnosis efficiency of the radiologists and Artificial Intelligence. The feasibility of using transfer learning in diagnosing Anterior Cruciate Ligament damage on the basis of the novel Enhanced MR DPP-TSO-Sag-FS-PDWI series was demonstrated, offering that it could assist radiologists in identifying ACL injuries, mostly grade 2. Transfer learning technique with pre-trained EfficientNet-B0 model was utilized, comprising ROI (Regions of Interest) and whole image inputs, to diagnose ACL automatically. The study included 235 people (90 women and 145 men, 37.91 ± 14.77 years), who had undergone both arthroscopic outcomes and DPP-TSO-Sag-FS-PDWI sequence. In detecting the common Anterior Cruciate Ligament (p = 0.063), there was not any statistical distinction between radiologists and the whole image, whereas the whole image outperformed the radiologists in injuries grade 2 and grade 1, which were, in turn, p = 0.003 and p = 0.012.

Kulwin et al.^24^ examined the comparison of the efficiency of the medical exams for ACL damage. In the present research, 133 patients with knee pathology were evaluated to determine the efficiency of the anterior drawer, Lachman, and lever tests in detecting ACL harm. 90 people of the patients were examined by arthroscopy, and 123 of them were examined by MRI. Then, the efficiency of the MRI and the tests were achieved. The study found significant differences in the accuracy of the lever test, the anterior drawer test, and the Lachman test in diagnosing ACL tears. The Lachman exam was the most sensitive, while the anterior drawer test was the least sensitive. The lever test had lower specificity compared to the Lachman and anterior drawer tests. All tears of ACL detected by the physical check were verified by Magnetic Resonance Image, and the MRI discoveries were consistent with discoveries of arthroscopic. There are medical uses of the anterior draw exam and Lachman test. However, the physicians should be so cautious, while interpreting the outcomes of the level test. Medical tests were extremely specific; however, their sensitivity were less than MRI.

Li et al.^25^ suggested a multimodal characteristic fusion model, utilizing deep learning model in order to investigate acceptance value of MRI (Magnetic Resonance Imaging) to detect ACL tears. The suggested algorithm was put to the test, and its performance was assessed. It was then employed to diagnose joint harms of knee. 30 patients with joint injuries of knee were picked. All the patients underwent detection through the use of a deep learning multimodal attribute fusion model based on arthroscopy and MRI. The study results revealed that the use of deep learning techniques in detecting sagittal MRI planes had a significant merit that its rate of accuracy was 96.28% in predicting ACL tear. Additionally, MRI showed specificity of 90.62%, accuracy of 92.17%, and a sensitivity of 96.78% in detecting ACL injuries. Finally, there was not significant discrepancy between these results and the ones accomplished via arthroscopy ($p>0.05$). Severe Anterior Cruciate Ligament patients with medial collateral ligament injury and bone contusion showed a significantly upper positive rate compared to those with chronic harm. Additionally, patients with chronic Anterior Cruciate Ligament damage and tear of meniscus or damage of cartilage had a substantially upper incidence compared to those with acute injury, indicating significant distinctions ($p<0.05$).

### Gap in the literature

Despite the progress made in diagnostic technologies, the accuracy of existing methods is still not ideal, resulting in frequent misdiagnoses that may have negative impacts on patient results. Research indicates a need for improved algorithms to analyze intricate knee MRI scans and identify ACL injuries with greater precision.

### The challenges of the previous works

The accurate and timely diagnosis of Anterior Cruciate Ligament (ACL) tears is a crucial obstacle in the realm of medical imaging. Prior studies have encountered difficulties in finding the right balance between computational efficiency and feature extraction accuracy. This often leads to the development of either a simplified model that disregards vital information or an excessively complex system that is not feasible for clinical application. Furthermore, effectively handling diverse and noisy datasets has proven to be a significant challenge.

### The main focus of the study and its novelty

The objective of this study is to close the existing gap by presenting an enhanced InceptionV4 model that utilizes the innovative Fractional-order Snow Leopard Optimization Algorithm (FO-SLO). Our research primarily concentrates on improving the feature extraction abilities of the InceptionV4 model, consequently enhancing the identification and diagnosis of ACL tears.

The proposed approach involves optimizing the InceptionV4 model, a widely used neural network architecture, using the Fractional-order Snow Leopard Optimization Algorithm (FO-LOA). By doing so, this study aims to effectively extract essential features from knee MRI images and enhance the detection of ACL tears. This new methodology promises to overcome the shortcomings of existing methods and enable accurate and prompt treatment interventions for patients.

Such timely interventions are critical for improving rehabilitation outcomes in patients with ACL injuries. The findings of this research offer promising potential for facilitating timely and accurate treatment interventions, ultimately contributing to better patient outcomes in ACL injury rehabilitation. Overall, this research is an important step forward in improving the accuracy of ACL tear detection and providing better care for patients. The main contributions of this paper can be highlighted as follows:

- A new method using an optimized InceptionV4 model for diagnosing ACL tears.
- Custom variant of the Snow Leopard Optimization Algorithm (FO-LOA) to apply InceptionV4.
- Demonstrating superior performance than the compared approaches.

## Preprocessing

Medical imaging that relies on deep learning is heavily dependent on preprocessing techniques, including noise reduction, contrast enhancement, and data augmentation, to optimize the analysis process. The application of deep learning algorithms to medical imaging involves training models to analyze and interpret MRIs. However, before inserting the picture into the deep learning systems, stages of pre-processing have been executed to enhance image quality and extract relevant features.

One critical aspect of preprocessing is noise reduction. Medical images often suffer from noise caused by factors such as acquisition processes or environmental interference. To address this, techniques such as Gaussian filtering^26^, median filtering^27^, or wavelet denoising^28^ are applied to reduce noise and improve image quality. By removing unwanted noise, the deep learning models can focus on extracting meaningful information from the images without being influenced by irrelevant distortions.

Another important preprocessing step is contrast enhancement. Enhancing image contrast plays a crucial role in improving the visibility of anatomical structures and abnormalities. Histogram equalization, adaptive contrast stretching, or other contrast enhancement algorithms are utilized to increase the dynamic range of pixel intensities. This helps to distinguish subtle details and features within the medical images, making them more suitable for accurate analysis by deep learning models.

Additionally, data augmentation techniques are employed to address the challenge of limited labeled datasets. Deep learning systems necessitate a large number of diverse data for effective training. However, acquiring such datasets take a lot of time and are challenging in the medical imaging field. Data augmentation overcomes this limitation by generating augmented versions of the available data through transformations such as rotation, translation, scaling, flipping, or adding noise. This artificially expands the diversity of the dataset of training, reduces overfitting, and enhances the ability of model for generalizing to unseen data. Data augmentation allows the deep learning models to learn from a wider range of variations and scenarios, enhancing their performance and generalization capability.

### Wang-Mendel-based Noise reduction

The Wang-Mendel-based noise reduction technique is a widely utilized method in image processing for the purpose of reducing noise and improving image quality. Its effectiveness is particularly noteworthy in the medical imaging field, where the preservation of critical details is essential for precise diagnosis and analysis.

The Wang-Mendel algorithm is founded on the principles of fuzzy logic and employs fuzzy membership functions to account for the inherent uncertainty and imprecision present in noisy images. The algorithm functions by assigning a degree of membership to each pixel, indicating the probability of it being affected by noise. Through the analysis of the local neighborhood of each pixel, the algorithm estimates the level of noise and adjusts the filtering process accordingly. The key steps involved in the Wang-Mendel-based noise reduction process are as follows:

#### Fuzzy membership function construction

- Denote the original noisy image as $X$ and the corresponding noise-free image as $Y$.
- Algorithm constructs Fuzzy membership functions to describe the degree of membership of each pixel in $X$ to the noisy class ($N$) and the noise-free class ($F$).
- The membership function for the noisy class is denoted as $\mu N(X)$ and for the noise-free class is denoted as $\mu F(X)$.
- These membership functions capture the statistical characteristics of both the noisy and noise-free image.

#### Noise level estimation

- The Wang-Mendel algorithm estimates the noise level in the image by evaluating fuzzy entropy.
- Fuzzy entropy measures the uncertainty associated with pixel intensities within a local neighborhood.
- The fuzzy entropy function is defined as $E\left(X\right)=-\int \int \mu F\left(X\right)log\left(\mu F\left(X\right)\right)dX+\int \int \mu N\left(X\right)log\left(\mu N\left(X\right)\right) dX$.
- By analyzing the uncertainty, the algorithm can estimate the noise level present in the image.

#### Adaptive filtering

- Based on the estimated noise level, adaptive filtering is performed to reduce noise while preserving image details.
- The strength of the filtering is controlled by fuzzy rules based on each pixel's degree of membership to the noisy class.
- The filtering process can be formulated as $Y=\eta \left(X, H\right)$, where $H$ is the filtering operator and $\eta$ is a fuzzy rule-based operator that adapts the filtering based on the membership values.

#### Post-processing

**Figure 1:**
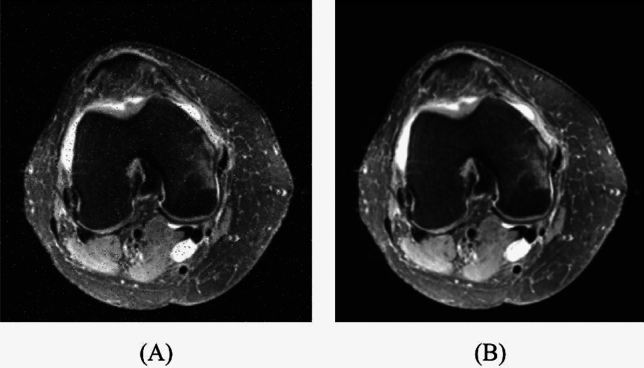
Wang-Mendel-based Noise reduction: (**A**) original image, and (**B**) denoised image.

- Contrast enhancement is used to improve the visual quality and diagnostic clarity of the image. Fig. (1) displays a sample for Wang-Mendel-based Noise reduction on ACL images.

### Contrast enhancement

Contrast enhancement in medical images is an essential requirement that can be used in the ACL input images. Tere are two types of contrast enhancement: direct and indirect techniques. Direct methods consist of applying a precise criterion for measuring the images contrast. Contrast enhancement is then performed by enhancing the identified criterion. The precise determination of visual contrast is a key endeavor in direct image quality enhancement. The direct contrast enhancement considers local and global information of the image, resulting in improved performance across a range of applications.

In contrast, the indirect contrast enhancement techniques are implemented based on the image histogram, so expanding the dynamic range of the grayscale values present in the image. This process leads to an improved contrast in the image. In recent years, there has been considerable interest in indirect approaches due to their clarity and straightforward presentation. These approaches can be classified into four distinct categories: image manipulation techniques, conversion-based methodologies, histogram adjustments, and approaches developed using artificial intelligence. The research reported in this study is based on histogram rectification methods, with a special focus on the use of the Historical Recurrence of Discrete Mean Rate (RMSHE) methodology.

The brightness of two balanced histograms ($BBHE$), is widely recognized as an initial solution to overcome the limitations of the HE technique. This approach additionally guarantees sufficient image brightness and improves contrast. The process entails partitioning the histogram into two distinct sub-histograms, utilizing the average brightness metric as the criterion for division. Subsequently, each partition is independently adjusted to achieve equilibrium. Let ${X}_{m}$ represent the value of mean in the image $X$. Given that ${X}_{m}$ falls within the range $\left[{X}_{0},{X}_{1},\dots ,{X}_{L-1}\right]$, the image of input has been divided into two sub-pictures, referred to as ${X}_{u}$ and ${X}_{L}$. The functions of transition in the sub-pictures are defined as follows:

1 $$f_{l} \left( X \right) = X_{0} + \left( {X_{m} - X_{o} } \right)D_{L} \left( X \right)$$

2 $${f}_{u}\left(X\right)={X}_{m+1}+\left({X}_{L-1}-{X}_{m+1}\right){D}_{U}\left(X\right)$$

where, ${D}_{U}\left(X\right)$ and ${D}_{L}\left(X\right)$ represent the relative density functions for ${X}_{U}$ and ${X}_{L}$, respectively.

The output image $BBHF$, is defined as follows:

3 $$Y={f}_{U}\left({X}_{L}\right)\cup {f}_{U}\left({X}_{U}\right)$$

**Figure 2:**
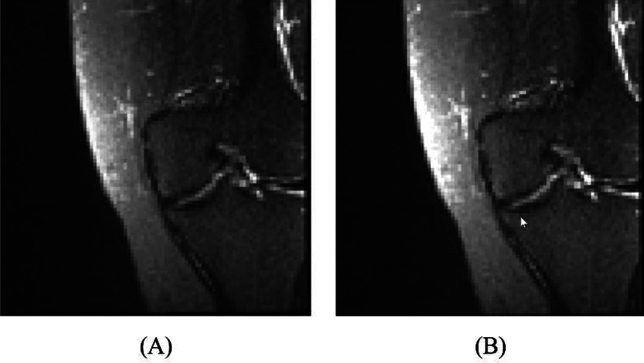
An instance of conducting the BBHE on an ACL image: (**A**) Input image and (**B**) image after enhancing.

Figure 2 demonstrates an instance of conducting the BBHE on an ACL image.

### Augmentation of data

The SMOTE (Synthetic Minority Over-Sampling Technique) is an oversampling technique that has been specifically designed to address class imbalance in machine learning datasets. Its primary objective is to generate synthetic samples for minority categories in imbalanced datasets. The aim is to balance the class distribution by increasing the representation of the minority class through the creation of artificial examples. The SMOTE algorithm follows a series of steps, which include the selection of a minority class sample, identification of k-nearest neighbors, creation of synthetic samples, and interpolation between samples. SMOTE generates new synthetic samples by multiplying the feature vector differences by a random value between 0 and 1, which is controlled by the user-defined parameter 'alpha'. By following this process, SMOTE increases the number of minority class instances, thereby addressing the imbalance in the dataset. This technique helps to ensure that the model learns from a more representative and balanced dataset.

The SMOTE method selects K-nearest neighbors for each sample ${x}_{i}$ in the subset ${S}_{min}$ of $S$, based on the Euclidean distance in an n-dimensional space. To generate artificial data samples, a random selection is made from the $K$-nearest neighbors. An accidental integer in the range of [0, 1] multiplies the difference between these two samples. This finding has been then augmented to the ${x}_{i}$ value. The resulting sample is positioned on the separative line between the two selected samples using the equation shown below:

4 $${Z}_{new}={z}_{i}+\gamma \times \left({z}_{j}-{z}_{i}\right)$$

where, $\gamma$ describes a random value in the range [0, 1].

## InceptionV4

### InceptionV4

The structure of the Inception-v4 as a convolutional neural network model was released in 2016 by researchers at Google^29^. Its major purpose was to address the complexity of the Inception-v3 model while keeping its remarkable performance on the ILSVRC 2015 challenge. Additionally, the Inception-v4 model addresses the possible integration of residual networks inside the Inception framework. This architecture contains two unique kinds of Inception structures: the Inception-ResNet architecture and the pure Inception architecture (referred to as Inception-v4).

**Figure 3:**
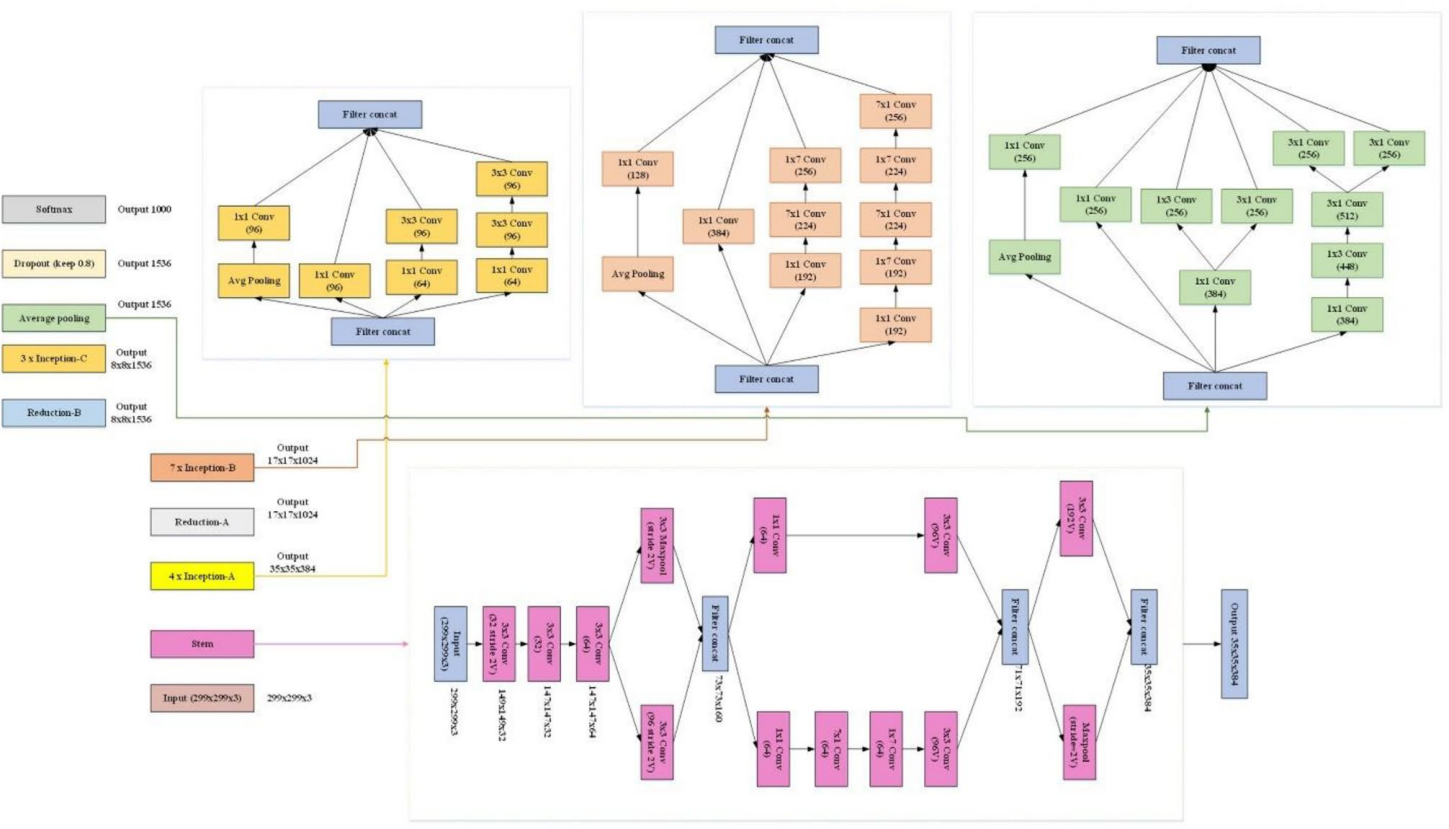
Arrangement of an Inception-v4.

To adjust the grid parameters, notably the height and width, the Inception-v4 model features configurable, Reduction Blocks. Compared to past incarnations, the architectural design of Inception-v4 demonstrates a greater degree of homogeneity. Particularly, unlike prior versions that needed partitioned copies to handle memory limits, Inception-v4 may be trained without the requirement for different replicas. This design exhibits a more homogenous structure and features a higher number of inception modules compared to its predecessors. Figure 3 indicates the arrangement of an Inception-v4.

The Inception-v4 network is a system that incorporates the utilization of Inception modules with residual connections. A complete mathematical depiction of the entire Inception-v4 network would require a significant amount of information and exceed the limits of a single solution ^30^. The Inception-v4 network, as depicted in Fig. 3, is composed of numerous Inception modules that were specifically designed to capture multi-scale properties through the use of parallel convolutional processes.

The Inception module employs various convolutional filters, including those with dimensions of 1 × 1, 3 × 3, and 5 × 5, to simultaneously examine input feature maps. The parallel pathways' outputs are concatenated along the channel dimension to combine the parallel paths. To reduce dimensionality before applying larger filters, 1 × 1 convolutions are also utilized, which reduces the computational burden.

As can be observed from Fig. 3, the Inception-v4 network is composed of many Inception modules that have been specifically built to capture multi-scale characteristics via the use of concurrent convolutional processes.

The design of the network incorporates Inception modules, which are strategically placed alongside average pooling and MaxPooling intermediary layers. In order to enhance the gradient flow during training, the Inception-v4 network utilizes residual connections, similar to the ResNet architecture^31^. A comprehensive mathematical description of the Inception-v4 model, including its precise structure, parameter dimensions, activation functions, and special layer layouts, can be found in the original study publication.

The structural data of ACL images can be taken into account when using this network for extraction of feature. Extraction of feature has been the systematic procedure of producing significant and practical representations from input data. The InceptionV4 model employed in this study is designed to automatically extract data from pictures using multiple layers.

The model comprises auxiliary classifiers, reduction blocks, global average pooling, fully connected layers, stem convolution layers, input modules, input images, fully connected layers, and an output layer with Softmax activation. Auxiliary classifiers are included at intermediary layers to regularize the network and collect fine-grained information at various resolutions. Reduction blocks periodically reduce the spatial dimensions of feature maps, maintaining a balance between spatial information and computational efficiency. Global average pooling has been utilized to convert spatial attribute maps into a fixed-length vector, capturing the most important characteristics while ignoring spatial data. The fixed-length feature vector is then connected to fully connected layers, followed by an output layer with Softmax activation for classifying the results.

### Objective function

However, Inception-v4 provides efficient results in solving the classification tasks, using developed optimizers for its structure can be helpful in decreasing its complexity and increasing its efficiency. In this study, to provide this case, an objective function based on the model loss value, classification error, and the number of its parameters has been introduced. By minimizing thus objective function, we can achieve better arrangement for our Inception-v4 model. The Objective function ($Obj$) is defined below:

5 $$Obj={\alpha }_{1}\times loss+{\alpha }_{2}\times {E}_{rate}+{\alpha }_{3}\times {N}_{param}$$

Such that, $loss$, ${N}_{param}$, and ${E}_{rate}$ represent the training loss, the total number of parameters, and the classification error rate in the Inception-v4 model, respectively, and the constants ${\alpha }_{i|i=\mathrm{1,2},3}$ are weighting factors to provide trade-off on the importance of components in the objective function such that $\sum {\alpha }_{i}=1$. Below is the mathematical equation for the aforementioned hyperparameters:

6 $$loss=-\sum \left(y\times {\text{log}}\left(\widehat{y}\right)\right)$$

7 $${N}_{param}=\sum \left(Number\,of\,parameters\,in\,each\,layer\right)$$

8 $${E}_{rate}=(incorrectly\,classified\,samples) / (total\,samples)$$

where, $\widehat{y}$ and $y$ defines, in turn, the predicted probability and the true label distribution.

In this study, a modified version of metaheuristic algorithm, namely Fractional-order Snow Leopard Optimization Algorithm is utilized for minimizing the aforementioned $Obj$ function. The idea is to optimal select of the decision variables, for $loss$, ${N}_{param}$ (the total number of parameters), ${E}_{rate}$ to provide the lowest value of the performance index. In the following, the designed developed metaheuristic has been explained in details. The main decision variables are number of parameters in each layer, and three coefficients, including ${\alpha }_{1}$, ${\alpha }_{2}$, and ${\alpha }_{3}$ as weighting factors. It should be noted that ${\alpha }_{1}+{\alpha }_{2}+{\alpha }_{3}=0$.

## Snow leopard optimization algorithm (SLOA)

In the current part, firstly, the individual has been presented. From that time on, according to modeling the individual’ natural activities and manners, a new optimality algorithm called the Snow Leopard Optimization Algorithm (SLOA) has been established. Numerical modeling of the suggested SLOA to resolve optimality problems has been offered.

### Snow leopard

The animal has been known as a kind of Panthera that lives in Central and South Asia’s tall mountains. Snow leopards are in high and mountainous regions that its height is in the range [3000, 4500] meters from the Himalayas, western China, southern Siberia and to the Tibetan Plateau, eastern Afghanistan, and Mongolia.

The fur of snow leopard is gray to white and has black spots on the upper side of his body, also, it owns longer rosettes on its back, and sides, and a hairy tail. The individual's belly is white. It has grey or light green eyes and large nasal cavities with a domed forehead and short muzzle. Its thick fur has hair between^5,12^ cm in length. Its legs are short, and its body is stocky, and a little smaller compared to other Panthera species of cats. The size of its should is 56 cm, and the size of its body is in the range [75, 150] cm. Tail of this animal has been extended between 80 to 105 cm. Its weight is in the range [22 ,55] kg, and in some cases big male weighs 75 kg, and a tiny female weighs less than 25 kg. It has doglike teeth that are 28.6 mm long; moreover, they are slenderer compared to other kinds of Panthera.

The animals possess diverse manners and leading, comprising the way they interact with other members, and the way they hunt and duplicate. These normal manners are utilized in the suggested SLOA’s model. In this study, we take advantage of simulating 4 optimization natural manners in the snow leopards’ life.

The first manner is travel paths and motion. Demonstrating the zig-zag outline motions of the animals as they transfer and pursue together creates a better examination of the solution space and overpass the optimized local regions.

The second manner is the way of hunting. Demonstrating the motions of the animals to hunt preys creates algorithm's convergence toward the optimization regions.

The third manner has been breeding. The animals’ breeding could be demonstrated as an integration of 2 participants of the group members that creates a novel member’s production that might enhance the efficiency of the algorithm in attaining optimal regions.

The 4th manner is a fatality. Demonstrating the fatality of powerless snow leopards creates the eradication of solutions and the algorithm’s unsuitable members. This would eradicate members in unsuitable regions from the search space. Further, modeling this manner makes the population stay steady throughout the several iterations.

### Mathematical modeling

In the suggested Snow Leopard Optimization Algorithm, every single individual is the member of population. In optimization algorithms, which are population-based, members of the population are recognized utilizing a matrix named the matrix of population. The rows’ quantity in the matrix of population has been considered the members’ quantity, and the columns’ quantity is the variables’ quantity in optimizing problem. The matrix of population is defined, which is demonstrated in the next formula.

9 $$Z = \left[ {\begin{array}{*{20}c} {Z_{1} } \\ \vdots \\ {Z_{i} } \\ \vdots \\ {Z_{N} } \\ \end{array} } \right]_{{N \times m}} = \left[ {\begin{array}{*{20}l} {z_{{1,1}} } \hfill \ldots \hfill {z_{{1,d}} } \hfill \ldots \hfill {z_{{1,m}} } \hfill \\ \vdots \hfill \ddots \hfill \vdots \hfill \ddots \hfill \vdots \hfill \\ {z_{{i,1}} } \hfill \ldots \hfill {z_{{i,d}} } \hfill \ldots \hfill {z_{{i,m}} } \hfill \\ \vdots \hfill \ddots \hfill \vdots \hfill \ddots \hfill \vdots \hfill \\ {z_{{N,1}} } \hfill \ldots \hfill {z_{{N,d}} \ldots } \hfill {z_{{N,m}} } \hfill {} \hfill \\ \end{array} } \right]$$

In which the snow leopard’s population is defined by $Z$, ${Z}_{i}$ has been the ${i}{th}$ individual, ${z}_{i}$ is the amount of ${d}{th}$ problem parameter proposed by ${i}{th}$ snow leopard, the individual's number has been determined by $N$, and $m$ is the quantity of problem variables.

Every single snow leopard’s situation that is a population member in resolving space of the problem defines the amounts of the parameters in problem. Consequently, for every single individual, an amount could be computed for the problem’s cost value. The cost value’s amount is indicated by a vector utilizing the following Equation.

10 $$P={\left[\begin{array}{c}{P}_{1}\\ \vdots \\ {P}_{i}\\ \vdots \\ {P}_{N}\end{array}\right]}_{N\times 1}=\left[\begin{array}{c}{P(Z}_{1})\\ \vdots \\ {P(Z}_{i})\\ \vdots \\ {P(Z}_{N})\end{array}\right]$$

where $P$ is the cost value’s vector and ${P}_{i}$ is the amount for the problem’s cost value on the basis of ${i}^{th}$ snow leopard.

the population members are renewed in the suggested SLOA on the basis of mimicking the snow leopards’ natural manners in 4 stages: movement, preying, breeding, and fatality. These 4 stages are modeled based on the mathematical formula and the stated natural manners are offered in the next parts.

#### Travel paths and motion

Similar to other cats, Snow leopards utilize odor marks to indicate the positions and move along the paths. These marks are generally made by scratching the earth with the back feet prior to putting scat. As well, the animals transfer in a zig-zag outline in unintended lines. Consequently, the animals could track together on the basis of this usual manner. The current stage has been calculated by the next formulas

$${z}_{i,d}^{F1}={z}_{i,d}+r\times \left({z}_{k,d}-I\times {z}_{i,d}\right)\times sign\left({P}_{i}-{P}_{k}\right) k\in \mathrm{1,2},3,\dots ,N$$

11 $$d=\mathrm{1,2},3,\dots ,m$$

12 $${Z}_{i}=\left\{\begin{array}{c}{Z}_{i}^{F1},{P}_{i}^{F1}<{P}_{i} \\ {Z}_{i}\end{array}\right.$$

13 $$I=round(1+r)$$

where, ${z}_{i,d}^{F1}$ is the novel amount for ${d}{th}$ problem variable obtained by ${i}{th}$ snow leopard on the basis of the first stage, $r$ is a stochastic number in ranging of 0 to 1, $k$ is the certain snow leopard’s row number for guiding ${i}{th}$ snow leopard in ${d}{th}$ axis, ${Z}_{i}^{F1}$ is the renew position of ${i}^{th}$ snow leopard on the basis of first stage, and ${P}_{i}^{F1}$ is its amount of cost value.

#### Preying

In the second stage of renewal the population member, the snow leopards’ manner throughout preying and assaulting the victim is utilized. The procedure and technique of preying on a snow leopard, according to the statement recorded in Hemis National Park, is that rocky cliffs are utilized by the snow leopard to shield itself when is close to its victim. Having reached a distance of forty meters from the victim, firstly, the snow leopard paced bit by bit for the 1st fifteen meters then ran the last twenty-five meters and lastly murdered by biting the victim’s neck.

The snow leopards’ natural manner throughout preying is determined by the next Equations. Equation (14) states the victim’s situation for ${i}^{th}$ snow leopard. Equation (15) mimics a snow leopard’s method of going toward its victim. Based on the explanations, the animal strides approximately 0.375 percent of the space to reach the prey and flees 0.625 percent of the space to grab the prey. Consequently, a $P$ has been utilized to mimic moves like this in Eq. (15). The proportion of the distance existing between the prey and the snow leopard that the individual strides, has been indicated by this parameter. In the recreation of the suggested SLOA, the value of the variable’s amount according to explanations is regarded as 0.375. In Eq. (16), the snow leopard's novel situation after the assault on the hunt is imitation. Accordingly, an efficient renewal is utilized that the novel situation is satisfactory to the member of an algorithm once the cost value amount in the novel situation is more proper than the former situation.

14 $$f_{i,d} = z_{j,d} , \,\,\,d = 1,2,3, \ldots ,m$$

15 $${z}_{i,d}^{F2}={z}_{i,d}+r\times (\left({f}_{i,d}-{z}_{i,d}\right)\times F+\left({f}_{i,d}-2\times {z}_{i,d}\right)\times \left(1-F\right))\times sign\left({P}_{i}-{P}_{f}\right)$$

16 $${Z}_{i}=\left\{\begin{array}{c}{Z}_{i}^{F2},{P}_{i}^{F2}<{P}_{i} \\ {Z}_{i}\end{array}\right.$$

where, ${f}_{i,d}$  is the ${d}^{th}$ dimension of the victim’s situation regarded for $ith$ snow leopard, ${P}_{f}$  is the cost value amount on the basis of the victim’s situation, *x* ${z}_{i,d}^{F2}$ is the novel amount for ${d}^{th}$ parameter of problem gained by ${i}^{th}$ individual based on the second stage, and ${P}_{i}^{F2}$ is its cost value amount.

#### Breeding

In this stage, on the basis of snow leopards’ natural breeding manner, novel members are equivalent to the whole population’s half that is added to the algorithm’s population. Actually, it has been supposed that a baby leopard would be born based on the coupling of two individuals. The snow leopard’s breeding procedure is mathematically calculated according to the stated concepts utilizing the next Equation.

17 $${C}_{l}=\frac{{z}_{l}+{Z}_{N-l+1}}{2} , \,\,\,l=\mathrm{1,2},3,\dots .\frac{N}{2}$$

where, ${C}_{l}$ is the ${l}^{th}$ cub that has been born from the coupling of two individuals.

#### Fatality

Creatures are always at the hazard of becoming extinct. Although breeding enhances the snow leopards’ population, the snow leopards’ number rests stable throughout the duplication because of deaths and fatalities. In the suggested Snow Leopard Optimization Algorithm, it has been supposed that the individuals will lose their life exactly as number of puppies that are born in every duplication after breeding. The principle of individual fatality in the Snow Leopard Optimization Algorithm has been the cost value amount. Consequently, snow leopards that own a powerless cost value are more likely to face death. Also, a number of born cubs might lose their life due to their owning weak cost value.

### The fractional‑order snow leopard optimization algorithm

While the Snow Leopard Optimization algorithm is a revolutionary meta-heuristic method with excellent results in optimization, it has encountered specific issues in particular circumstances. In the present study, the results of an additional modification proposed to fix the algorithm's flaws are more accurate and worldwide. This advancement makes use of a fractional change. For using the fractional approach, a short-term introduction of FC (Fractional Calculus) has been supplied. The Fractional-order Calculus (FC) is recently proposed as a useful tool for improving the performance of meta-heuristic algorithms ^32^. The FC technique proposed a well-organized assessment of the process, memory, and inherited traits. As a result, the FC is a valuable tool for improving the performance of meta-heuristic algorithms by considering memory during solution renewal. The Grunwald–Letnikov (GL) model is a popular FC model. The following is the mechanism:

18 $${S}^{\sigma }\left({z}_{i,d}\left(t\right)\right)=\underset{h\to 0}{{\text{lim}}}\frac{1}{{h}^{\sigma }}{\sum }_{a=0}^{\infty }{\left(-1\right)}^{a}\left(\genfrac{}{}{0pt}{}{\sigma }{a}\right){z}_{i,d}\left(t-ah\right)$$

where,

19 $$\left(\genfrac{}{}{0pt}{}{\sigma }{a}\right)=\frac{\Gamma (\sigma +1)}{\Gamma (a+1)\Gamma (\sigma -a+1)}=\frac{\sigma \left(\sigma -1\right)\left(\sigma -2\right)\dots (\sigma -a+1)}{a!}$$

Such that, $\Gamma \left(t\right)$ specifies the gamma function, and the $GL$ fractional derivative of order $\sigma$ is determined by ${D}^{\sigma }\left({z}_{i,d}\left(t\right)\right)$ that can be evaluated by the following:

20 $${S}^{\sigma }\left[{z}_{i,d}\left(t\right)\right]=\frac{1}{{T}^{\sigma }}{\sum }_{a=0}^{N}\frac{{\left(-1\right)}^{a}\Gamma \left(\sigma +1\right){z}_{i,d}\left(t-aT\right)}{\Gamma \left(a+1\right)\Gamma \left(\sigma -a+1\right)}$$

The duration of memory (memory window) is denoted by $N$, while the sampling time is determined as $T$. The derivative order operator is represented by $\sigma$. When $\sigma$ is assigned a value of 1, the previous equation is transformed into the following equation:

21 $${S}^{1}\left[{z}_{i,d}^{1}\left(t\right)\right]={z}_{i,d}^{F1}\left(t+1\right)-{z}_{i,d}\left(t\right)$$

22 $${S}^{1}\left[{z}_{i,d}^{2}\left(t\right)\right]={z}_{i,d}^{F2}\left(t+1\right)-{z}_{i,d}\left(t\right)$$

where ${S}^{1}\left[{z}_{i,d}\left(t\right)\right]$ describes the difference between the 2 following actions.

Here, the FC mechanism is used to enhance the leopard optimization algorithm’s situation as follows:

23 $${z}_{i,d}^{F1}-{z}_{i,d}=r\times \left({z}_{k,d}-I\times {z}_{i,d}\right)\times sign\left({P}_{i}-{P}_{k}\right)$$

24 $${z}_{i,d}^{F2}-{z}_{i,d}=r\times (\left({f}_{i,d}-{z}_{i,d}\right)\times F+\left({f}_{i,d}-2\times {z}_{i,d}\right)\times \left(1-F\right))\times sign\left({P}_{i}-{P}_{f}\right)$$

And the formula can be finally achieved as :

25 $${S}^{1}\left[{z}_{i,d}^{1}\left(t\right)\right]={\sum }_{a=1}^{m}\frac{{\left(-1\right)}^{a}\Gamma \left(\delta +1\right){z}_{i,d}^{1}\left(t+1-a\right)}{\Gamma \left(a+1\right)\Gamma \left(\sigma -a+1\right)}$$

26 $${S}^{1}\left[{z}_{i,d}^{2}\left(t\right)\right]={\sum }_{a=1}^{m}\frac{{\left(-1\right)}^{a}\Gamma \left(\delta +1\right){z}_{i,d}^{2}\left(t+1-a\right)}{\Gamma \left(a+1\right)\Gamma \left(\sigma -a+1\right)}$$

Therefore, the total formulation of Eq. (23) and Eq. (24) can be rewritten as follows:

27 $${z}_{i,d}^{F1}={\sum }_{a=1}^{m}\frac{{\left(-1\right)}^{a}\Gamma \left(\delta +1\right){z}_{i,d}^{2}\left(t+1-a\right)}{\Gamma \left(a+1\right)\Gamma \left(\sigma -a+1\right)}+r\times \left({z}_{k,d}-I\times {z}_{i,d}\right)\times sign\left({P}_{i}-{P}_{k}\right)$$

28 $${z}_{i,d}^{F2}={\sum }_{a=1}^{m}\frac{{\left(-1\right)}^{a}\Gamma \left(\delta +1\right){z}_{i,d}^{2}\left(t+1-a\right)}{\Gamma \left(a+1\right)\Gamma \left(\sigma -a+1\right)}+r\times (\left({f}_{i,d}-{z}_{i,d}\right)\times F+\left({f}_{i,d}-2\times {z}_{i,d}\right)\times \left(1-F\right))\times sign\left({P}_{i}-{P}_{f}\right)$$

Utilizing the given equation and considering m = 4 (representing the first four terms of the memory data), the Snow Leopard 's situation can be updated using the following formula:

29 $${z}_{i,d}^{F1}=\frac{1}{1!}\sigma {z}_{i,d}\left(t\right)+\frac{1}{2!}\sigma \left(1-\sigma \right){z}_{i,d}\left(t-1\right)+\frac{1}{3!}\sigma \left(1-\sigma \right)\left(2-\sigma \right){Z}_{i}\left(t-2\right)+\frac{1}{4!}\sigma \left(1-\sigma \right)\left(2-\sigma \right)\left(3-\sigma \right){z}_{i,d}\left(t-3\right)+r\times \left({z}_{k,d}-I\times {z}_{i,d}\right)\times sign\left({P}_{i}-{P}_{k}\right)$$

30 $${z}_{i,d}^{F2}=\frac{1}{1!}\sigma {z}_{i,d}\left(t\right)+\frac{1}{2!}\sigma \left(1-\sigma \right){z}_{i,d}\left(t-1\right)+\frac{1}{3!}\sigma \left(1-\sigma \right)\left(2-\sigma \right){Z}_{i}\left(t-2\right)+\frac{1}{4!}\sigma \left(1-\sigma \right)\left(2-\sigma \right)\left(3-\sigma \right){z}_{i,d}\left(t-3\right)+r\times (\left({f}_{i,d}-{z}_{i,d}\right)\times F+\left({f}_{i,d}-2\times {z}_{i,d}\right)\times \left(1-F\right))\times sign\left({P}_{i}-{P}_{f}\right)$$

### Flowchart of SLOA

In the suggested FO-SLO algorithm, snow leopards are renewed in every single repetition based on the first and second stages, therefore the algorithm’s population according to the third and fourth stages are confronted with normal procedures of breeding and fatality. The paces are reiterated till the cease situation is touched. Once executing the Snow Leopard Optimization Algorithm on an optimality problem has been completed, the algorithm provides the most optimal solution available.

**Figure 4:**
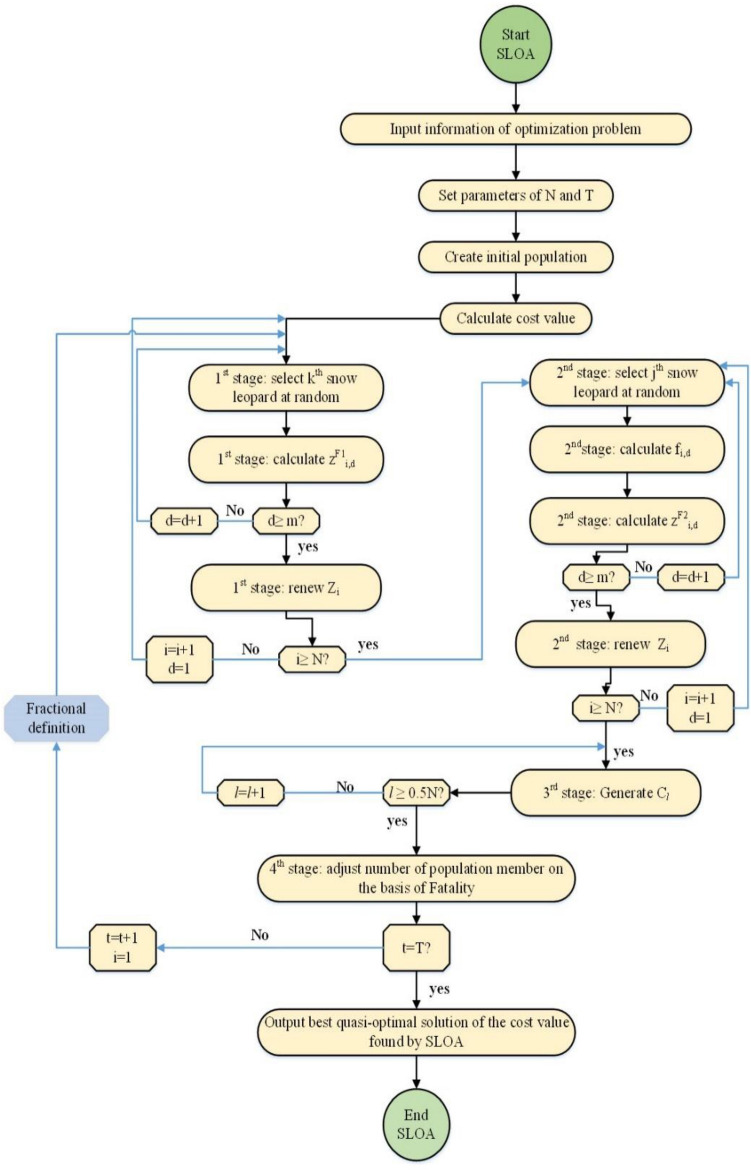
The flowchart diagram of the proposed FO-SLO algorithm.

The Fractional-order Snow Leopard Optimization Algorithm (FO-SLOA) was likely selected by the authors for various reasons. This nature-inspired metaheuristic algorithm emulates the hunting behavior of snow leopards and has demonstrated effectiveness in solving different optimization problems by offering quasi-optimal solutions that are closer to the global optimum. The fractional calculus component of the FO-SLOA enables it to leverage memory properties and intermediate processes, thereby enhancing the convergence and steady-state performance of the algorithm (see Fig. 4).

The benefits of utilizing the FO-SLOA include its capacity to deliver more suitable solutions and its competitive performance when compared to similar algorithms. Furthermore, it is believed that the FO-SLOA is capable of effectively managing the complexities associated with optimizing neural network structures like InceptionV4, which is essential for accurately extracting features from medical images. The decision not to opt for Particle Swarm Optimization (PSO) or Grey Wolf Optimizer (GWO) may be attributed to various factors. Although PSO and GWO are popular optimization algorithms, each algorithm possesses its own set of strengths and weaknesses depending on the specific problem being addressed.

The FO-SLOA may have been discovered to offer a superior balance between exploration and exploitation, or it may have been deemed more appropriate for the unique characteristics of the medical image analysis problem being addressed by the authors. Additionally, the utilization of fractional calculus in the FO-SLOA could potentially provide a distinct advantage in handling the non-linear and high-dimensional nature of the optimization problem in medical imaging.

Comparative studies involving PSO, GWO, and other algorithms have demonstrated that no single algorithm consistently outperforms the others in all scenarios, and the selection often relies on the specific requirements and nature of the problem at hand. Hence, it is possible that the authors conducted preliminary experiments to determine the most suitable optimizer for their study. The performance of the FO-SLOA in these experiments, particularly its efficient ability to navigate complex landscapes and find optimal solutions, likely influenced their decision-making process.

### The proposed network

**Figure 5:**
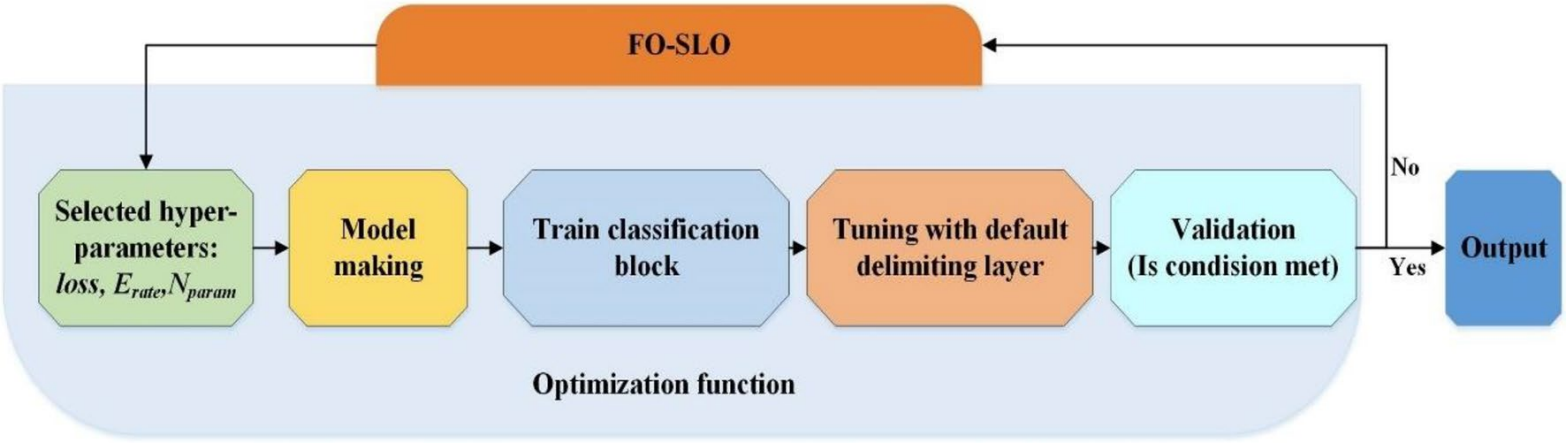
Principal architecture of the FO-SLO-optimized InceptionV4.

As stated before, the proposed FO-SLO is then applied to the InceptionV4 to optimize its hyperparameter values and produce an optimal architecture. This is illustrated in Fig. 5.

The FO-SLO approach was used to optimize the InceptionV4 network's main design, which is shown in Fig. 5. The FO-SLO technique attempts to determine the InceptionV4 model's ideal set of hyperparameters, producing an architecture that can improve the model's performance.

It is believed that the enhanced InceptionV4 architecture will demonstrate greater capabilities and efficiency in comparison to the original version thanks to the use of the FO-SLO approach. This graphic gives a visual picture of how the FO-SLO optimization method impacted the design. Table 1 lists the hyperparameters, their range, and the ideal value that the FO-SLO in this research chosen for the categorization of ACL injuries.

**Table 1:** Hyperparameters, their range, and the ideal value that the FO-SLO in this research chosen for the categorization of ACL injuries.

Parameter	Lower range	Upper range	Weight
Loss	0	–	0.5
Error rate	0	1	0.7
Num parameters	0	–	0.2
$\alpha$	0	1	1/3
$\beta$	0	1	1/3
$\gamma$	0	1	1/3

The results show that the error rate is given a weight of 0.7, indicating that it is significant for the fitness function. The loss has a weight of 0.5, indicating the prominence of the loss in the efficiency of optimization. The weight given to the number of parameters is 0.2, indicating that while it is not as important, it should still be taken into account when managing the model's difficulty.

## Dataset description

In this study, we utilized MRNet Dataset for our analysis. The MRNet Dataset is composed of 1,370 knee Magnetic Resonance Image tests conducted at Stanford University Medical Center. The data contain 1,104 unusual tests, 508 tears of meniscal and with 319 Anterior Cruciate Ligament tears. The brands were accomplished by physical extraction from medical reports. The dataset was collected from the Stanford University School of Medicine, which outlines the terms and conditions for using the MRNet dataset ^33^. The dataset might be copied from the link below: https://stanfordmlgroup.github.io/competitions/mrnet/

**Figure 6:**
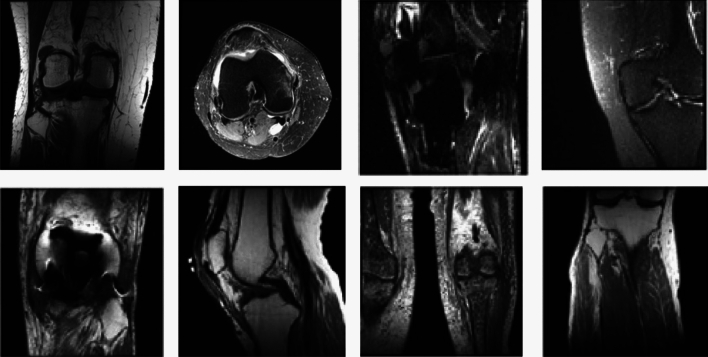
A collection of samples taken from the MRNet dataset.

This study separates the examinations into two dissimilar sets. The first set, referred to as the training set, Comprises 959 investigations, which accounts for 70% of all data. The remaining 30% of data, consisting of 411 examinations, have been designated as the test data. To ensure the inclusion of a minimum of 50 positive instances for each label category, namely meniscal tear, abnormal, and ACL tear, in each set, a stratified random sampling technique was employed to create the training and tuning sets. Furthermore, the examinations conducted on each patient were consolidated into a single dataset. Figure 6 displays a collection of samples taken from the MRNet dataset.

It is evident that Fig. 6 showcases a compilation of sample instances procured from the MRNet dataset. The MRNet dataset encompasses diverse examples that are illustrated in the aforementioned figure.

## Results and discussions

This paper introduced an improved version of the InceptionV4 model as a unique way to identify Anterior Cruciate Ligament (ACL) injuries. The approach used in this study involves the utilization of a modified version of the Snow Leopard Optimization Algorithm, namely the Fractional-order Snow Leopard Optimization Algorithm (FO-LOA), for the purpose of extracting crucial characteristics from knee MRI scans. The study suggests that the proposed methodology provides better rehabilitation outcomes by enabling healthcare professionals to make more informed decisions about the most suitable treatment plans for each patient.

The utilization of the FO-LOA optimization algorithm is a key aspect of this research work. By incorporating this algorithm into the design of the InceptionV4 model, the researchers provide an enhanced framework for feature extraction from knee MRI images. The effectiveness of the FO-LOA optimization algorithm in improving the accuracy of ACL tear detection demonstrates its potential as a valuable tool for medical image analysis. Overall, this research highlights the significance of an optimized InceptionV4 model combined with the FO-LOA optimization algorithm for diagnosing ACL tears. By significantly improving the accuracy of ACL tear detection and offering enhanced precision, this approach has the potential to support healthcare professionals in making timely and informed treatment decisions, ultimately leading to improved rehabilitation outcomes for patients with ACL injuries.

This dataset has been distributed into 2 subsets, with the set of training, comprising 85% of the information and the exam set, including 15% of the information. The dataset comprises of ACL MRI images obtained from individuals with both healthy and those ACL tears. The research used technology implementations to aid in the detection of ACL injuries. The experiment was conducted using Matlab R2019b programming language and have been done on a high-speed processing system (High Computing Performance) consists of several computing clusters and the integration between them leads to centralized management of tasks. Hardware specifications: 2 nodes, 2 graphics cards and 8 × 16GB of memory, NVIDIA® Tesla K80 GPUs 2 × Intel® Xeon® E5-2695 v3 @ 2.30 GHz processor.

### Algorithm validation

For ensuring a reliable assessment of the suggested FO-SLO algorithm, this section presents a comparative analysis of the FO-SLO algorithm in relation to various contemporary and popular approaches, including the Firefly Algorithm, Bat Algorithm, Water Wave Algorithm, Whale Optimization, and Salp Swarm Algorithm. The evaluation of these algorithms was conducted on the CEC2017 benchmarks. The effectiveness of these algorithms was measured using average accuracy (AVG) and standard deviation (STD). The algorithm's consistency was assessed utilizing the consistency index of algorithm. It is worth noting that the formulae for benchmark functions F21-F23 remain consistent throughout the 23 traditional benchmark functions, thus only the first 21 functions were utilized. The assigned values for the variables utilized in the studies are presented in Table 2.

**Table 2:** Assigned values for the variables utilized in the studies.

Algorithm name	Parameter setting
Firefly algorithm (FA)	Alpha: 0.5, Beta: 1, Gamma: 1, I0: 1
Bat algorithm (BA)	A: 0.9, R: 0.95, Q_min_: 0, Q_max_: 2
Water wave algorithm (WWA)	Amplitude: 1, Frequency: 1, Speed: 1
Whale optimization (WO)	A: 2, A_min_: 0, C: 1, b: 1, l: 0.02, p: 0
Salp swarm algorithm (SSA)	C1: 2, C2: 2, W: 0.7, k: 3, m: 10

In order to provide a fair and impartial comparison between the proposed FO-SLO algorithm and the other algorithms mentioned, a uniform population size of 60 and a maximum iteration of 120 have been selected for all algorithms under consideration. Tables 2 and 3 demonstrate that the FO-SLO algorithm exhibits superior performance in solving 19 functions along with the maximum accuracy of average, as observed in the tests conducted on the changed classic performance index. The average results are presented in Table 3.

**Table 3:** Comparison average results based on the studied algorithms.

Function	FO-SLO	FA	BA	WWA	WO	SSA
F1	1.37	1630.56	2860.88	4325.06	1468.53	2330.75
F2	8.92	11.82	950,634.48	14.58	598.69	4,152,286,093.34
F3	709.51	2949.47	7206.26	8608.19	2558.45	3168.81
F4	2.29	2.21	6.89	2.64	1.98	3.76
F5	23.14	3247.93	31,513.77	5337.74	880.21	4518.46
F6	1.03	1002.31	5437.62	1968.69	1548.47	1905.62
F7	0.00	4.37	9.76	1.63	0.35	3.68
F8	548.96	672.71	473.47	787.81	627.68	507.34
F9	19.39	12.30	17.68	12.65	11.30	16.26
F10	0.81	1.45	0.25	0.41	0.70	0.19
F11	0.05	22.66	15.96	18.70	16.12	22.41
F12	0.32	0.26	2.83	0.70	0.30	0.99
F13	0.03	0.65	2.80	1.32	1.60	1.30
F14	1.46	1.56	1.79	1.50	1.52	1.21
F15	0.00	0.00	0.01	0.01	0.00	0.00
F16	0.00	0.00	0.00	0.01	0.00	0.00
F17	0.00	0.00	0.20	0.06	0.00	0.01
F18	0.00	0.00	8.90	4.83	0.00	0.44
F19	0.00	0.00	0.63	0.39	0.00	0.15
F20	0.30	0.26	0.30	0.23	0.18	0.37
F21	1.73	2.05	0.19	1.63	1.34	1.64

As observed from the results of the CEC2017 benchmark function experiments, FO-SLO demonstrates the highest accuracy in solving 25 functions. Subsequently, the stability of the algorithms will be assessed by analyzing the STD values. Table 4 illustrates the juxtaposition of STD outcomes based on the investigated algorithms.

**Table 4:** Comparison STD results based on the studied algorithms.

Function	FO-SLO	FA	BA	WWA	WO	SSA
F1	2.65	21.82	21.38	8.68	8.71	17.27
F2	3.34	23.65	34.32	53.85	16.17	8.50
F3	2884.62	10,638.83	36,503.14	29,070.00	8896.97	35,593.17
F4	12.70	33.53	57.29	36.73	17.29	28.12
F5	33.01	49.32	63.33	119.15	206.02	56.06
F6	5.41	24.20	27.66	90.85	27.89	18.72
F7	0.00	8.49	41.98	7.53	0.93	7.63
F8	100.07	93.54	330.41	110.47	167.12	304.66
F9	40.54	188.07	325.92	115.04	112.59	177.27
F10	2.53	4.12	8.75	14.42	10.27	7.63
F11	0.75	20.56	28.54	10.49	24.34	210.36
F12	0.15	1.40	9.80	4.21	1.66	5.69
F13	0.02	1.44	8.55	3.55	2.83	5.01
F14	1.28	0.97	2.42	2.50	1.03	0.82
F15	0.00	0.00	0.01	0.01	0.00	0.00
F16	− 0.78	− 0.78	− 0.41	− 0.60	− 0.54	− 0.69
F17	0.35	0.27	0.29	0.22	0.21	0.21
F18	1.67	2.13	11.20	3.92	1.64	2.72
F19	− 2.23	− 2.04	− 1.46	− 1.30	− 2.63	− 1.73
F20	− 1.15	− 1.49	− 1.08	− 1.32	− 0.82	− 1.13
F21	− 4.92	− 4.14	− 0.43	− 3.67	− 4.74	− 3.13

**Figure 7:**
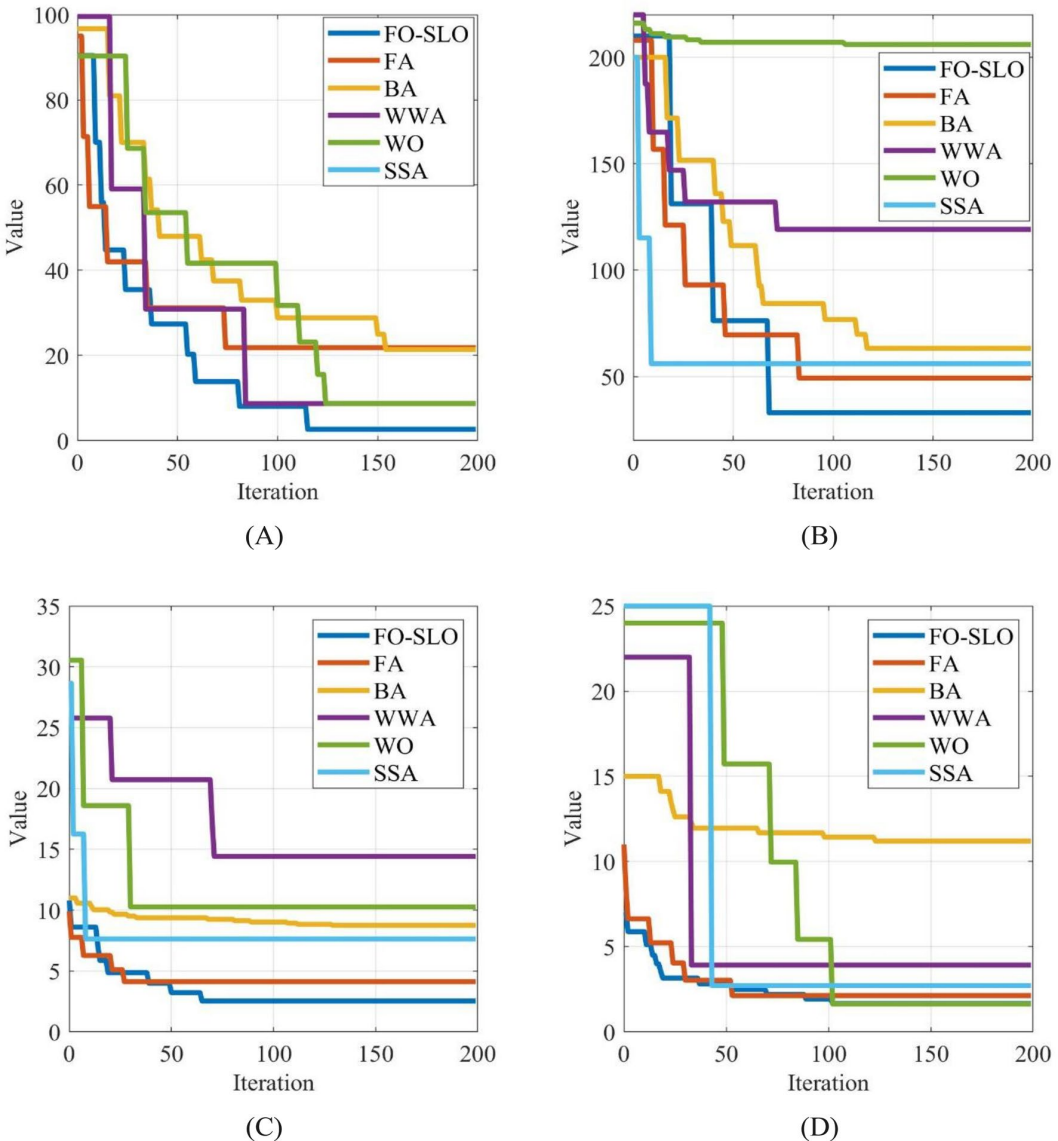
The convergence analysis of the proposed FO-SLO in comparison with different algorithms for (**A**) f1, (**B**) f5, (**C**) f10, (**D**) f18.

On most benchmark functions, the FO-SLO has the maximum accuracy. Compared to other algorithms, the FO-SLO possesses an advantageous measure of stability. In the tables presented in this study, the calculated standard deviation values for the ultimate accuracy of resolving CEC2017 performance indices and changed classical performance indices are displayed. A small SD of accuracy suggests the model performs satisfactorily while solving the exam purpose repeatedly for $N$ iterations, thereby indicating that FO-SLO is stable. Figure 7 shows the convergence analysis for some functions for more clarification.

The convergence analysis graph displays various algorithms or methods, including FO-SLO, FA, BA, WWA, WO, and SSA. These methods are plotted to showcase the decrease in their values as iterations progress. This graph plays a vital role in comparing the efficiency and speed of these methods in achieving a lower value, which is often the objective in optimization problems. Based on the description, it appears that the proposed FO-SLO exhibit faster convergence towards a lower value, indicating its higher efficiency. By visually comparing the graph, one can select the most suitable method for a specific problem based on its convergence speed and stability.

### Model simulation results

To ascertain the effectiveness of the preprocessing stage on the ACL images processing, the methodology was implemented in two conditions. In one condition, we assume that we have not applied any preprocessing, and in the second one, we have considered that we provided preprocessing on the system.

For evaluating the efficacy of the suggested model, six conventional metrics, namely Accuracy (Acc), Precision (Prc), Sensitivity (Sns), Specificity (Spc), F-Measure (F1), and Matthews Correlation Coefficient (MCC) were employed. The ensuing formulae can be utilized to compute these indicators:

31 $$Acc=\frac{TP+TN}{TP+TN+FP+FN}$$

32 $$Prc=\frac{TP}{TP+FP}$$

33 $$Sns=\frac{TP}{TP+FN}$$

34 $${\text{F}}1=\frac{2\times Prc\times Sns }{Prc+Sns}$$

35 $$Spc=\frac{TN}{\text{TN+FP}}$$

36 $$MCC=\frac{TP\times TN-TP\times FN}{\sqrt{\left(TP+FP\right)\times \left(TP+FN\right)\times \left(TN+FP\right)\times \left(TN+FN\right)}}\times 100$$

where, $TP$ and $TN$ describe, in turn, true positive, and true negative, and $FN$ and $FP$ represent, in turn the false negative and false positive.

**Figure 8:**
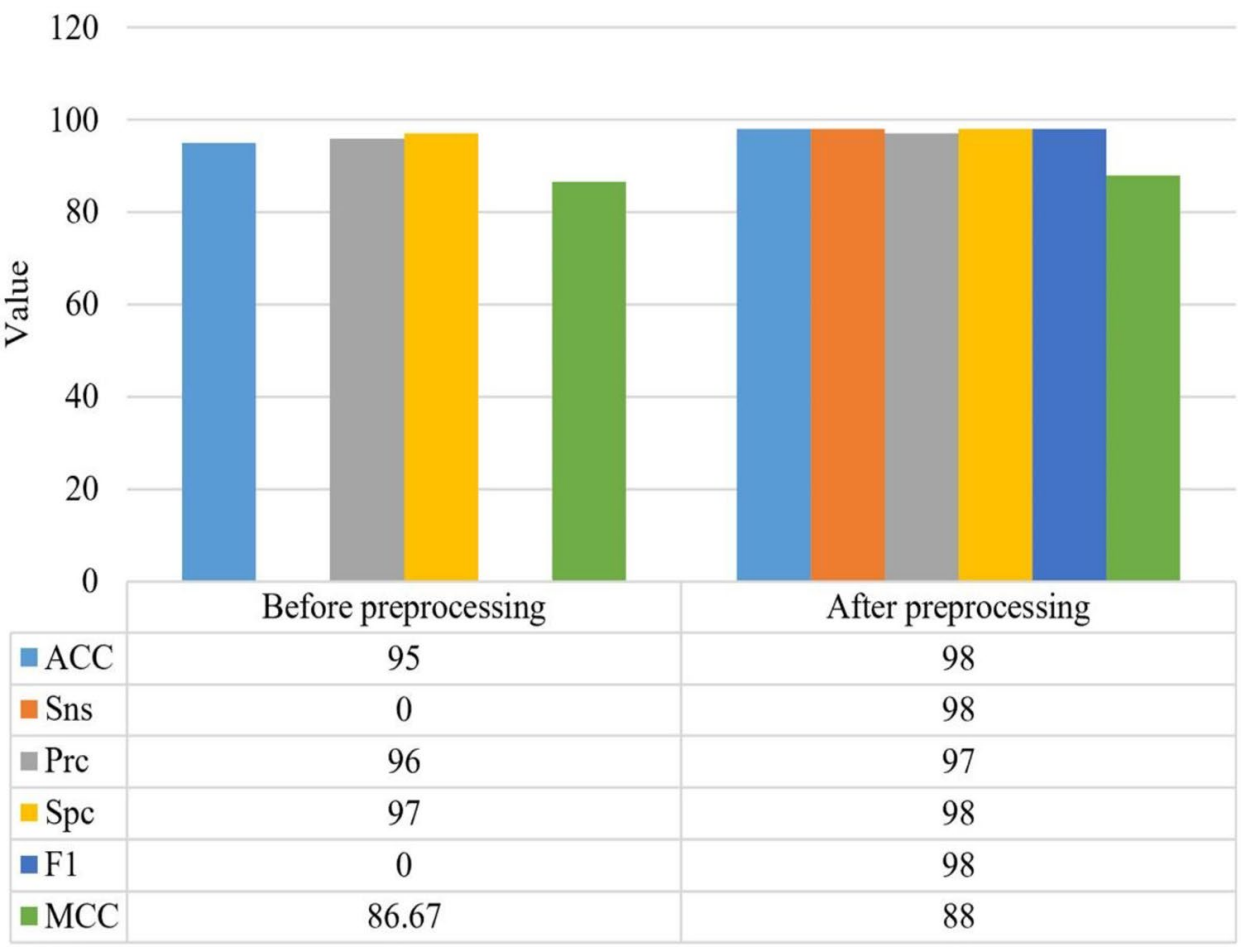
Findings of the ACL tear dataset identification, both before and after preprocessing.

The findings of the ACL tear dataset identification, both before and after preprocessing, are shown in Fig. 8.

In cases where there is a zero division, the term “0” is used to indicate that the result is not applicable or relevant. As can be observed from the results, before preprocessing of the ACL images, the method achieved an accuracy of 95.00%. Unfortunately, the sensitivity value is not available (“0”) due to the presence of zero division, indicating that the result is not relevant in this case. The precision achieved was 96.00%, and the specificity was 97.00%. The F1 score is not provided (NA) in this case. After preprocessing, the method's performance improved significantly. The accuracy increased to 98.00%, indicating a more accurate overall classification. The sensitivity and precision both remained at 98.00%, suggesting that the method effectively identified positive instances and maintained a high level of precision. The specificity also improved to 98.00%, demonstrating improved identification of negative instances. Furthermore, the F1 score reached 98.00%, indicating a well-balanced trade-off between precision and sensitivity. The MCC value after preprocessing was 88.00%, showing a strong correlation between the predicted and observed classes.

After proofing the effectiveness of using the preprocessing in our work, we need to validate it more through the outcomes with some other modern approaches. In this study, the outcomes are compared with Convolutional Neural Network (CNN) ^34^, Inception-v3 ^35^, Deep Belief Networks and Improved Honey Badger Algorithm (DBN/IHBA) ^22^, integration of the CNN with an Amended Cooking Training-based Optimizer version (CNN/ACTO) ^36^, Self-Supervised Representation Learning (SSRL) ^37^.

In this study, simulations were performed utilizing the MRNet dataset to evaluate the classification accuracy of the analyzed procedures. The results of the categorization examination of the suggested model, compared to other modern deep models on the MRNet dataset, are presented in Table 5.

**Table 5:** Categorization examination of the suggested system compared to other modern deep systems on the MRNet dataset.

Method	Acc	Sns	Prc	Spc	F1	MCC
Proposed method	98.00	98.00	97.00	98.00	98.00	88.00
DBN/IHBA^22^	81.06	79.67	70.71	88.34	79.48	71.54
SSRL^37^	75.68	72.95	76.36	88.80	90.44	89.66
Inception-v3^35^	77.37	79.68	90.19	84.14	89.07	86.92
CNN^34^	93.51	78.67	76.85	95.35	79.35	78.48
CNN/ACTO^36^	89.30	90.43	91.51	83.61	92.26	85.92

By comparison, the other contemporary deep models exhibited differing degrees of performance. In general, the proposed approach surpassed the other modern deep designs on the MRNet dataset with regards to accuracy, sensitivity, precision, F1 score, and MCC. This implies that the proposed model may provide superior performance and enhanced classification capability when compared to existing models for the specified dataset.

## Discussions

Within this segment, we explore an in-depth conversation regarding the research study centered on improving the InceptionV4 model through the utilization of the Fractional-order Snow Leopard Optimization Algorithm (FO-SLOA) for the detection of Anterior Cruciate Ligament (ACL) tears. We assess the obtained outcomes, contrast them with current methods, and emphasize the unique contributions of this study.

The study's outcomes demonstrate the exceptional performance of the FO-SLOA-optimized InceptionV4 model on the MRNet dataset. Key numerical indicators include an accuracy rate of 98.00%, sensitivity of 98.00%, precision of 97.00%, specificity of 98.00%, F1-score of 98.00%, and a Matthews Correlation Coefficient of 88.00%. These metrics underscore the model's capacity to precisely categorize ACL tear cases while minimizing false positives and ensuring accurate identification of non-ACL cases. The preprocessing procedures, such as Wang-Mendel-based noise reduction, BBHE contrast enhancement, and SMOTE data augmentation, significantly contribute to the model's success by improving feature extraction from knee MRI images.

When compared to current methods, the FO-SLOA-optimized InceptionV4 surpasses other models commonly utilized in medical image analysis. Evaluating against models like DBN/IHBA, SSRL, Inception-v3, CNN, and CNN/ACTO reveals the superior performance of the proposed model across various metrics. The innovation of the study lies in the FO-SLOA, a fusion of fractional calculus and snow leopard-inspired optimization, providing benefits like memory-inclusive search, effective navigation of complex optimization landscapes, and exploration of global optima crucial for precise feature extraction.

The clinical implications of the research are substantial, with potential advantages for clinical practice. Accurate detection of ACL tears allows for timely interventions, reducing patient risk and enhancing rehabilitation outcomes for individuals with ACL injuries. The study's results not only showcase the effectiveness of the proposed model but also pave the way for future advancements in medical image analysis and diagnostic accuracy.

## Conclusions

The ACL has been considered a significant ligament within the joint of knee, serving to provide stability and prevent excessive forward movement of the tibia in relation to the femur (thighbone). ACL tears are a common injury resulting from sports-related activities, which can occur due to sudden stops, changes in direction, or direct impact to the knee. The prompt and accurate diagnosis of ACL tears is crucial in determining appropriate treatment strategies and minimizing the risk of further complications. The utilization of deep learning and metaheuristics techniques offers several advantages in the diagnosis of ACL injuries. Deep learning algorithms, mostly CNNs, excel at automatically extracting meaningful features from medical images. By training CNN models on large datasets of knee MRI images, these models can learn to identify patterns and characteristics indicative of an ACL tear. Deep learning-based approaches have demonstrated superior accuracy in detecting ACL tears compared to traditional image analysis techniques. This research study presents an optimized version of the InceptionV4 model, which utilizes a modified metaheuristic algorithm, namely Fractional-order Snow Leopard Optimization Algorithm (FO-LOA) to detect ACL tears. The model optimized with FO-LOA was subjected to thorough testing on the MRNet dataset, demonstrating exceptional performance metrics. It achieved an accuracy rate of 98.00%, sensitivity of 98.00%, precision of 97.00%, specificity of 98.00%, F1-score of 98.00%, and a Matthews Correlation Coefficient (MCC) of 88.00%. These outcomes surpass well-known methodologies like Convolutional Neural Network (CNN), Inception-v3, Deep Belief Networks, and Improved Honey Badger Algorithm (DBN/IHBA), as well as the combination of CNN with a modified Cooking Training-based Optimizer version (CNN/ACTO) and Self-Supervised Representation Learning (SSRL). This progress represents a significant advancement in ACL injury diagnosis. The use of FO-SLO to optimize the InceptionV4 framework shows promise in improving the accuracy of ACL tear detection, enabling prompt and efficient treatment interventions.

## Data Availability

All data generated or analysed during this study are included in this published article.
